# Identification of stiffness-induced signalling mechanisms in cells from patent and fused sutures associated with craniosynostosis

**DOI:** 10.1038/s41598-017-11801-0

**Published:** 2017-09-13

**Authors:** Sara Barreto, Arlyng González-Vázquez, Andrew R. Cameron, Fergal J. O’Brien, Dylan J. Murray

**Affiliations:** 1 Tissue Engineering Research Group (TERG), Department of Anatomy, Royal College of Surgeons in Ireland (RCSI), Dublin 2, Ireland; 2 Trinity Centre for Bioengineering, Trinity College Dublin (TCD), Dublin 2, Ireland; 3 Advanced Materials and Bioengineering Research (AMBER) Centre, CRANN Institute, Trinity College Dublin, Dublin 2, Ireland; 4 National Paediatric Craniofacial Centre, Temple Street Children’s University Hospital, Dublin 1, Ireland

## Abstract

Craniosynostosis is a bone developmental disease where premature ossification of the cranial sutures occurs leading to fused sutures. While biomechanical forces have been implicated in craniosynostosis, evidence of the effect of microenvironmental stiffness changes in the osteogenic commitment of cells from the sutures is lacking. Our aim was to identify the differential genetic expression and osteogenic capability between cells from patent and fused sutures of children with craniosynostosis and whether these differences are driven by changes in the stiffness of the microenvironment. Cells from both sutures demonstrated enhanced mineralisation with increasing substrate stiffness showing that stiffness is a stimulus capable of triggering the accelerated osteogenic commitment of the cells from patent to fused stages. The differences in the mechanoresponse of these cells were further investigated with a PCR array showing stiffness-dependent upregulation of genes mediating growth and bone development (TSHZ2, IGF1), involved in the breakdown of extracellular matrix (MMP9), mediating the activation of inflammation (IL1β) and controlling osteogenic differentiation (WIF1, BMP6, NOX1) in cells from fused sutures. In summary, this study indicates that stiffer substrates lead to greater osteogenic commitment and accelerated bone formation, suggesting that stiffening of the extracellular environment may trigger the premature ossification of the sutures.

## Introduction

**Figure 1:**
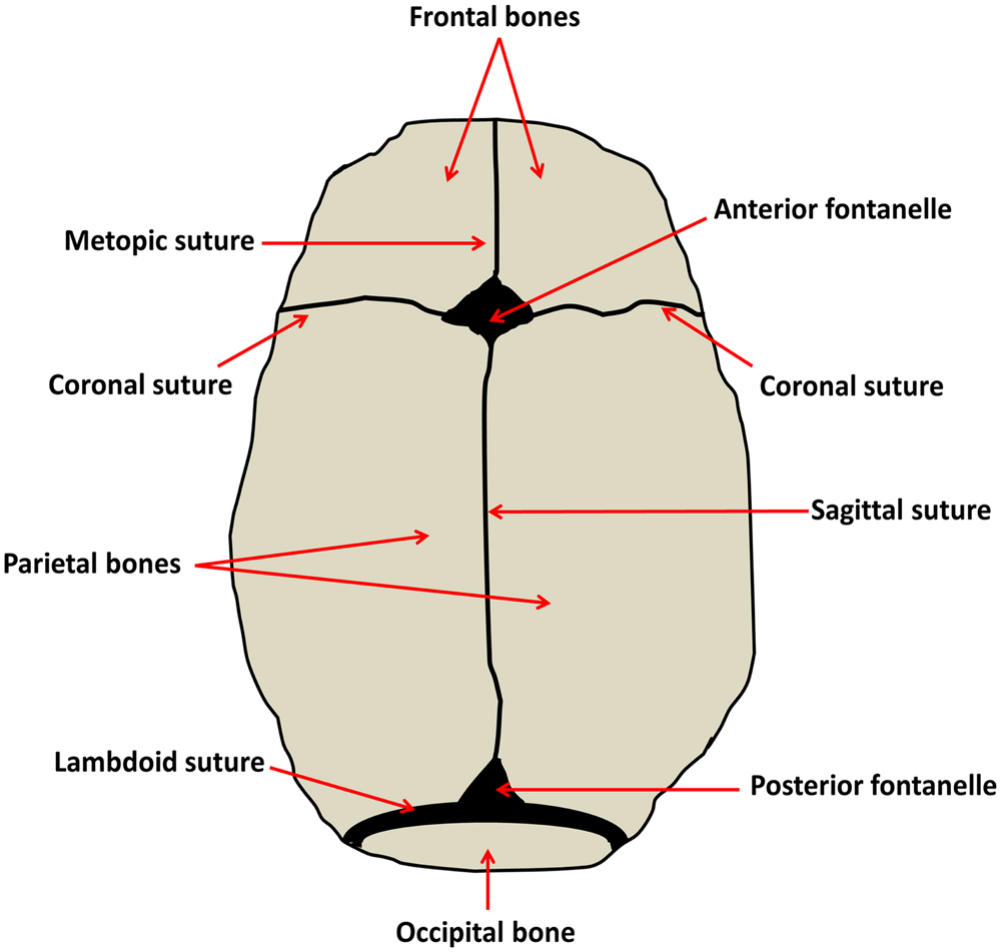
Schematic representation of the top view of the normal skull of a newborn.

Human calvarial bones are derived from paraxial mesoderm and craniofacial neural crest cells and are mostly formed by intramembranous ossification^1, 2^. Sutures are fibrous joints in the vertebrate skull. They separate the skull bone plates and are essential for the expansion and subsequent growth of the skull. They contain two osteogenic fronts and intervening fibrous tissue. The osteogenic fronts are primary sites of osteogenesis mediating much of the growth of the face and skull vault^3^. The sutures consist of non-ossified mesenchymal tissue with several cell lineages such as mesenchymal cells, fibroblast-like cells, osteogenic cells and osteoclasts^4, 5^. The advancing osteogenic fronts at the edges of the suture of the flat calvarial bones and provide a niche for highly proliferative osteogenic progenitors that express early markers of osteogenic differentiation, which may proliferate or differentiate in a tightly regulated program orchestrated through appropriate molecular cues to enable the growth of the skull^6, 7^. Sutures are very flexible, which is a key mechanical property to allow for deformation of the skull during childbirth and subsequent skull and brain growth during development^8, 9^. There are several sutures separating the six bony plates of the skull (Fig. 1), including: the metopic suture separating the frontal bones along the midline; the sagittal suture separating the parietal bones; the left and right halves of the coronal suture separating the frontal and parietal bones and; the left and right halves of the lambdoid suture separating the parietal bones from the single occipital bone posteriorly^2^. During normal development, sutures remain patent (unfused) until adulthood, with the exception of the metopic suture that undergoes fusion during the first months of life^10^. When mature or fusing, sutures are distinguished by well-developed fibre systems that not only unite the calvarial bones but also act to resist deformation in compression and tension^9^.

Calvarial bone formation and suture development can sometimes be altered in developmental diseases, such as craniosynostosis, which is caused by an acceleration of ossification within patent sutures of the skull, which prematurely fuse restricting brain growth during development. Craniosynostosis can be classified as syndromic (associated with consistent extracranial dysmorphisms of the face, trunk, or extremities) or non-syndromic^11, 12^. Important pathways for suture development and closure have been already identified, including the finding that alterations of the MSX2 expression^6, 13^, FGFRs-1, -2, -3 and TWIST have been associated with craniosynostosis^2, 14–17^. However, these studies typically identify genetic mutations rather than changes in the levels of gene expression. In general, the genes contributing to craniosynostosis can be categorised as genes encoding molecules that effect osteogenic upregulation, osteoclastogenic downregulation, cell patterning, extracellular matrix, apoptosis, cell proliferation, or vascular function^7, 18, 19^. While most studies on the genetic mechanism associated with craniosynostosis are described for syndromic cases, non-syndromic craniosynostoses -in addition to potential genetic causes- are believed to have strong environmental causes, including changes in the biomechanical forces^4, 15, 18, 20, 21^.

Of particular relevance to this study, the influence of biophysical factors on the changes in ossification observed during craniosynostosis has yet to be investigated. Sutures do not have intrinsic growth potential and therefore, they produce new bone at the sutural edges of the bone fronts in response to external stimuli, such as signals arising from the expanding neurocranium and from the dura mater, cyclic loading from muscle activity and traumatic impacts^5, 20, 22^. Therefore, transmission of altered mechanical forces at the cranial sutures during development may increase the risk of non-syndromic craniosynostosis and affect the physiological process of osteogenesis in the sutures^23^. It has been demonstrated that mechanical strain applied to the sutures can cause changes in cell size and number, vascularisation, changes in suture morphology and upregulation of osteogenic markers, including alkaline phosphatase (ALP) and osteopontin (OPN)^24^. Also, Oppenheimer *et al*. has demonstrated that cyclical forces led to premature fusion of sagittal sutures^25^ but the effect of changes in the stiffness of the cranial suture tissues in bone formation has yet to be investigated. Since bone generation is mediated by signalling mechanisms that include stimuli from the surrounding environment, pathological changes in the physiology of the sutures of children with craniosynostosis may be associated with changes in the stimuli provided by the extracellular environment that impairs the functional capacity of cells within the sutures to sense and respond to these stimuli. In order to understand if changes in the substrate stiffness are associated with premature fusion of sutures and the specific mechanotransductive mechanisms that underpin this response, we cultured cells isolated from patent and fused sutures of non-syndromic children diagnosed with craniosynostosis on soft and stiff collagen-coated polyacrylamide substrates. In doing so, we aimed to elucidate differences in the molecular signalling pathways and in the behaviour of osteoblastic cells from patent and fused sutures and whether these differences are driven by changes in the stiffness of the microenvironment.

## Results

### Cells from fused sutures have higher osteogenic potential than cells from patent sutures

**Figure 2:**
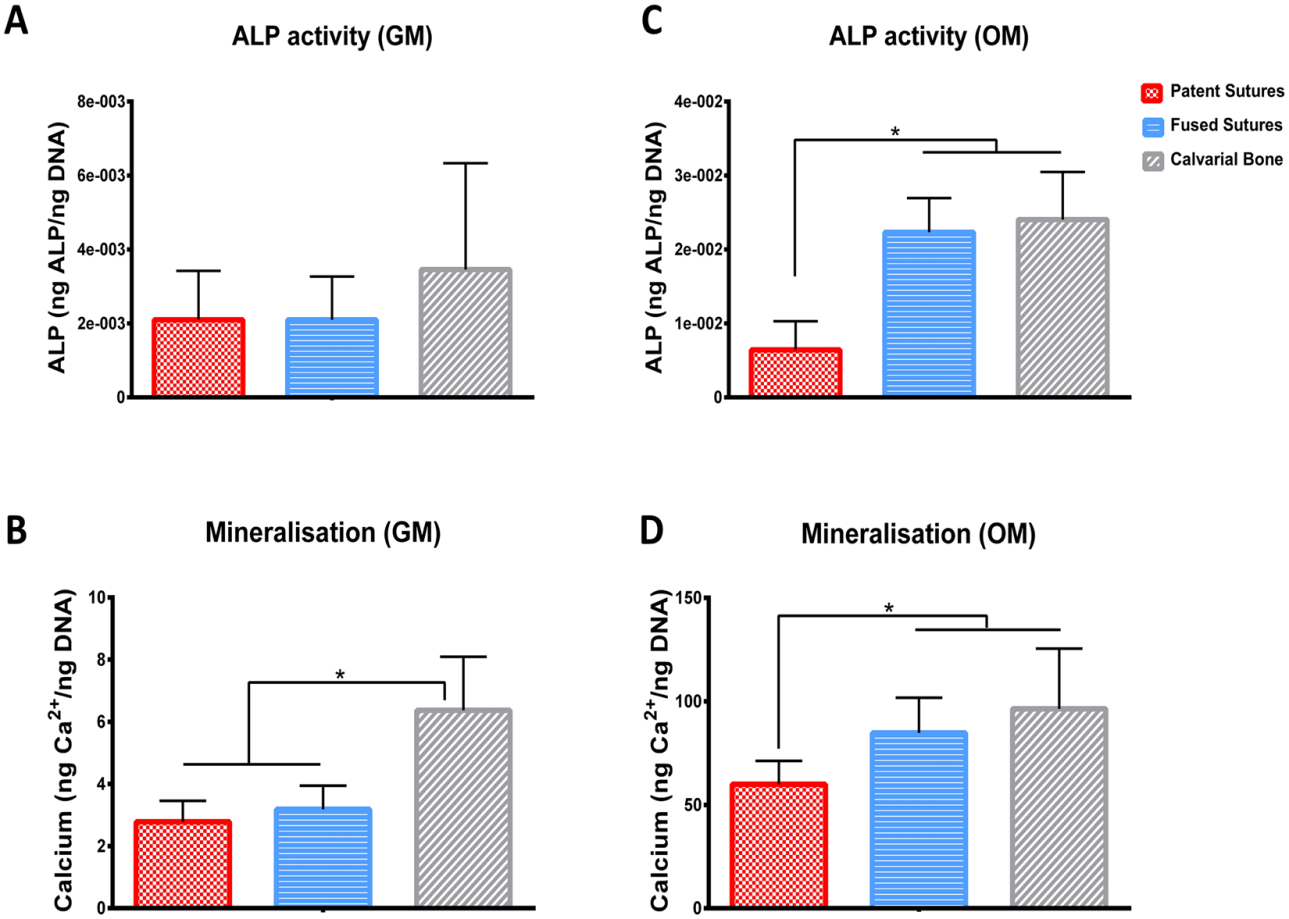
Characterisation of the osteogenic potential of cells from patent and fused sutures by means of measurement of alkaline phosphatase (ALP) activity and mineralisation in comparison with cells from normal calvarial bone used as a control for fully differentiated osteoblasts. ALP activity of cells from patent and fused sutures cultured in (**A**) growth medium (GM) and in (**B**) osteogenic medium (OM) after 7 days in culture. Mineralisation of cells from patent and fused sutures in (**C**) GM and in (**D**) OM after 14 days in culture measured by means of calcium deposition. Donors N = 3; Technical repeats n = 3; *p < 0.05.

**Figure 3:**
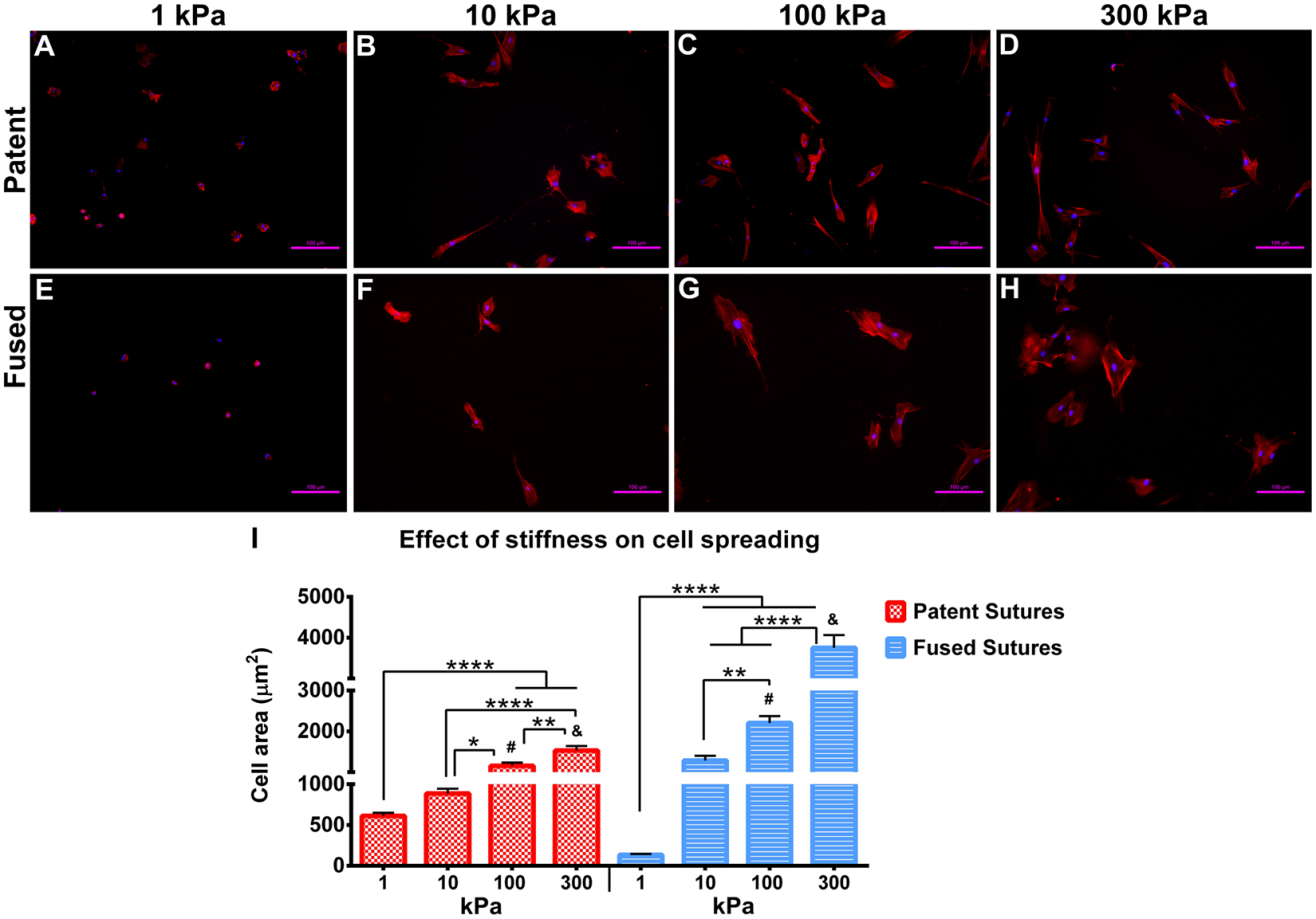
Effect of stiffness in the spreading area of cells from patent and fused sutures. Morphology of cells from patent sutures attached to the collagen-coated polyacrylamide substrates of (**A**) 1 kPa, (**B**) 10 kPa, (**C**) 100 kPa and (**D**) 300 kPa. Morphology of cells from fused sutures attached to the collagen-coated polyacrylamide substrates of (**E**) 1 kPa, (**F**) 10 kPa, (**G**) 100 kPa and (**H**) 300 kPa. (**I**) Measurement of the area of cells from patent and fused sutures spread after 48 hours of culture on substrates of different stiffness and in growth medium. Scale bar 100 µm. Donors N = 3; n = 21. *p < 0.05; **p < 0.01; ^#,&,^****p < 0.0001.

In order to evaluate the intrinsic osteogenic potential of cells isolated from patent and fused sutures of children with craniosynostosis, we analysed their potential for bone formation in both growth and osteogenic media and measured the expression of osteogenic markers, including ALP activity and calcium deposition. Cells from patent sutures expressed similar levels of ALP activity (Fig. 2A) as cells from fused sutures when cultured in growth medium after 7 days in culture. Similar levels of mineralisation (Fig. 2B) were obtained in cells from patent and fused sutures after 14 days in culture in growth medium, which were lower than cells from normal calvarial bones (p < 0.05), used as a control for fully differentiated osteoblasts. When cultured in osteogenic medium, cells from fused sutures expressed higher ALP activity on day 7 (Fig. 3C) and higher calcium release at day 14 (Fig. 3D) compared to cells from patent sutures (p < 0.05). In osteogenic medium, ALP activity and calcium release of cells from fused sutures was similar to the levels obtained from cells from the normal calvarial bones, indicating that cells from fused sutures are able to maturate faster towards the osteogenic phenotype than cells from patent sutures.

### Stiff substrates promote higher spreading area in cells from fused sutures than in cells from patent sutures

Numerous biophysical properties of the cellular microenvironment have been shown to influence the behaviour of cells^26–28^. In particular, substrate stiffness has been shown to modulate cell morphology, cytoskeletal structure, and adhesion of several cell types^29^. Here, we analysed the effect of substrate stiffness on the morphology of cells isolated from the patent (Fig. 3A–D) and fused (Fig. 3E–H) sutures of children with non-syndromic craniosynostosis in order to understand more about the mechanosensitivity of these cells. Results showed a 2.5- and a 1.7-fold increase in the spread area of cells from patent sutures cultured on stiffer 300 kPa substrates compared to those cultured on 1 kPa (p < 0.05) and 10 kPa substrates (p < 0.01), respectively (Fig. 3I). A 2.9-fold increase was observed in the spreading area of cells from fused sutures cultured on 300 kPa substrates compared to those cultured on 10 kPa substrates (p < 0.05). The spreading area of cells from patent sutures does not vary significantly from the spreading area of cells from fused sutures when cultured on substrates of low (1 and 10 kPa) stiffness (Fig. 3I). However, cell spreading area was higher in cells from fused sutures than in cells from patent sutures cultured on stiff (100 and 300 kPa) substrates (p < 0.001). On 100 and 300 kPa substrates, cells from patent sutures are more spindly and smaller (Fig. 3C,D) than cells from fused sutures in the same substrates. In turn, the latter are bigger and more rounded (Fig. 3G,H) resembling differentiated osteoblasts. The 10 and 300 kPa substrates, representing soft and stiff environments respectively, were further used for the analysis of the mechanosensitivity of cells from patent and fused sutures as well as for the analysis of the stiffness effect on osteogenic differentiation.

### Stiffness-dependent increase in mineralisation by cells from fused sutures

**Figure 4:**
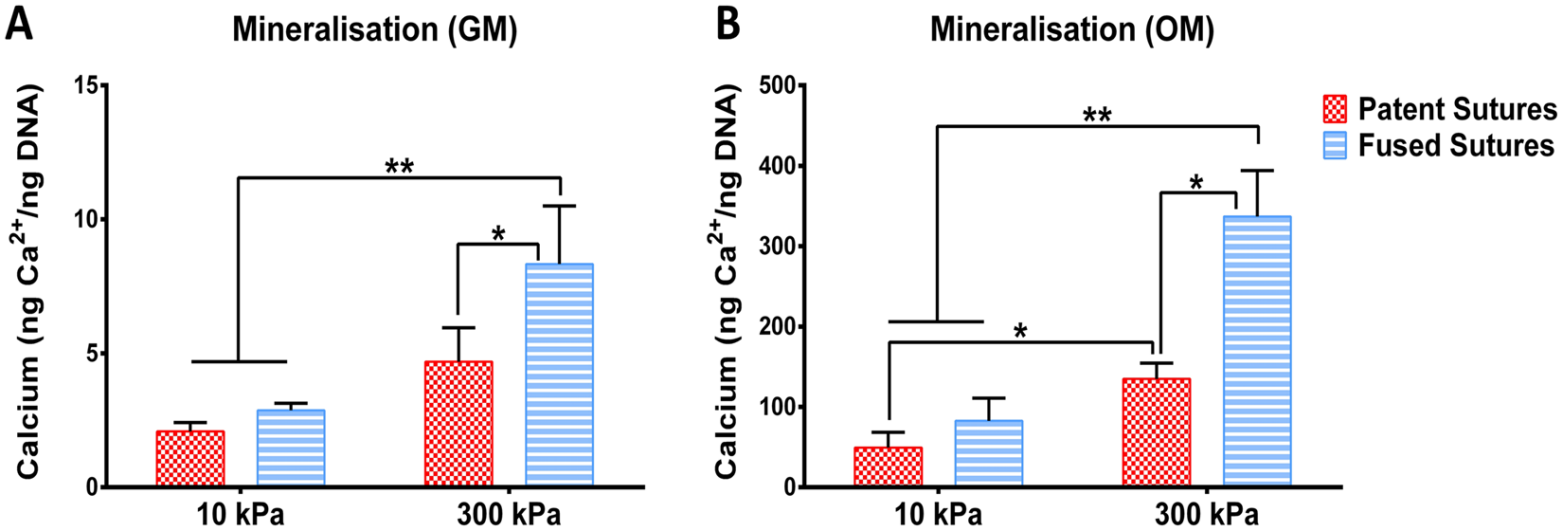
Stiffness-dependent mineralisation in cells from patent and fused sutures. Mineralisation of cells from patent and fused sutures measured by means of calcium deposition on 10 kPa and 300 kPa substrates in (**A**) growth medium (GM) and in (**B**) osteogenic medium (OM) after 14 days in culture. Donors N = 3; Technical repeats n = 3; *p < 0.05; **p < 0.01.

In order to understand whether the stiffness-dependent osteogenic potential of cells from patent sutures is different from fused sutures, we cultured these cells on collagen-coated polyacrylamyde substrates of 10 kPa (soft) and 300 kPa (stiff) and analysed calcium deposition after 14 days in culture in both growth and osteogenic medium. In growth medium (Fig. 4A), stiffness did not have an effect on the mineralisation capacity of cells from patent sutures (i.e., from 10 kPa to 300 kPa). Conversely, in GM, calcium deposition by cells from fused sutures cultured on 300 kPa substrates was significantly higher (p < 0.01) than the calcium levels produced by cells from both patent and fused sutures cultured on 10 kPa substrates, indicating that stiffness has a greater effect on the osteogenic commitment of cells from fused sutures, even in the absence of soluble induction factors. Additionally, when cultured on 10 kPa substrates in growth medium, no statistical differences were observed in the calcium deposition between cells from patent and fused sutures. When cultured on 300 kPa substrates in growth medium, the calcium deposition of cells from patent sutures was lower than the calcium deposition by cells from fused sutures (p < 0.05). In osteogenic medium (Fig. 4B), there is a stiffness-dependent increase in the mineralisation of cells from both patent (p < 0.05) and fused sutures from 10 to 300 kPa (p < 0.01). This indicates that, other than chemical cues, biophysical cues in the form of substrate stiffness also increase the osteogenic potential of not only cells from fused sutures but also of cells from patent sutures. These results indicate that stiff environments induce higher levels of mineralisation in cells from fused sutures than in cells from patent sutures.

### Stiffness-dependent upregulation of genes is differentially expressed between cells from fused and patent sutures

**Figure 5:**
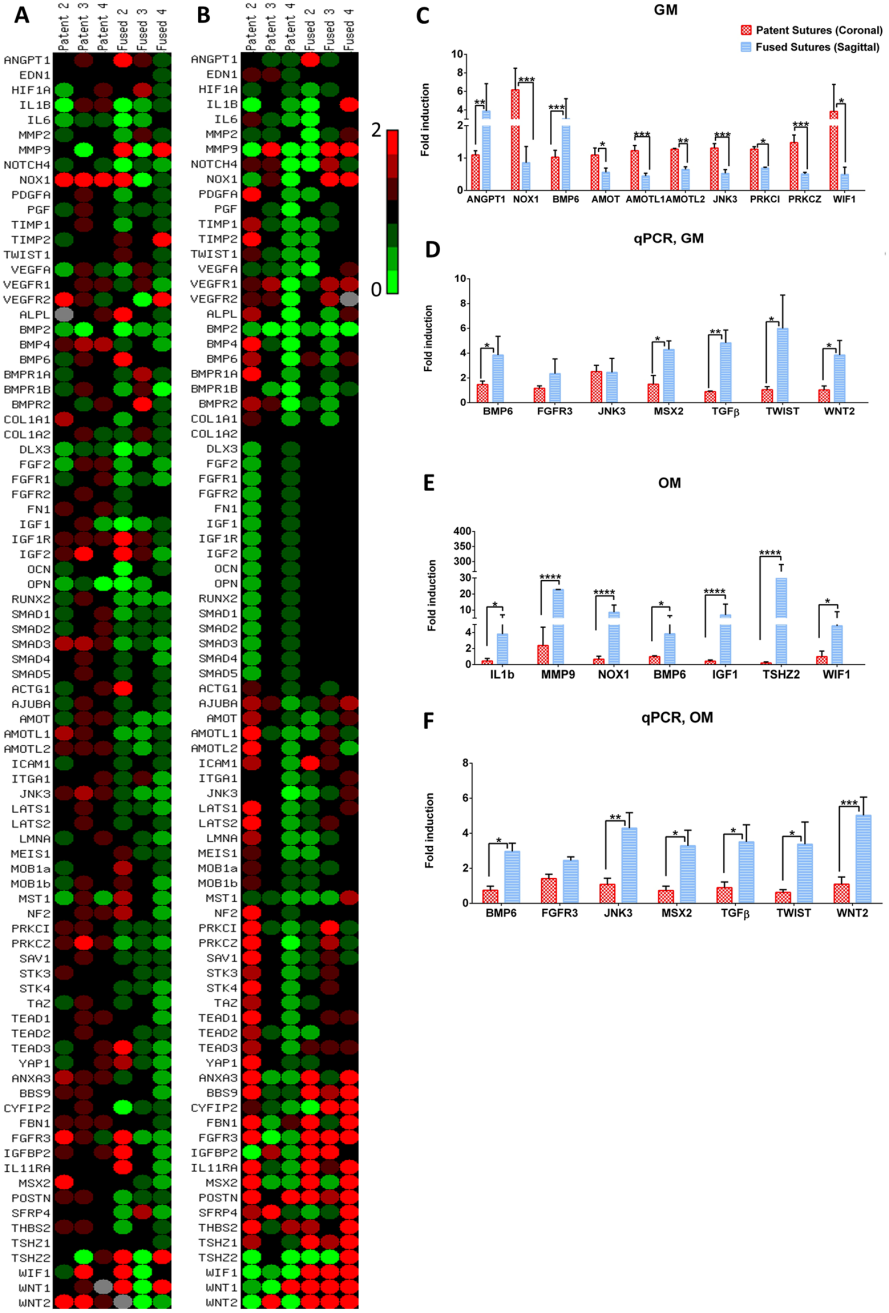
Stiffness-induced gene upregulation of cells from fused and patent sutures cultured for 7 days on soft (10 kPa) and stiff (300 kPa) substrates. (**A**) Heatmaps of the genetic expression of cells from patent and fused sutures representing the fold induction from cells cultured with growth medium (GM) on stiff to soft substrates, per donor. (**B**) Heatmaps of the genetic expression of cells from patent and fused sutures representing the fold induction from cells cultured with osteogenic medium (OM) on stiff to soft substrates, per donor. (**C**) Statistically significant stiffness-dependent gene upregulation of cells from fused and patent sutures cultured in GM and presented as the average fold induction of cells cultured on stiff and soft substrates. (**D**) Selection of genes validated by qPCR comparing fold induction from stiff to soft of cells from fused and patent sutures cultured in GM. (**E**) Statistically significant stiffness-dependent gene upregulation of cells from fused and patent sutures cultured in OM and presented as the average fold induction between the genetic expressions of cells cultured on stiff and soft substrates. (**F**) Selection of genes validated by qPCR comparing fold induction from 300 kPa to 10 kPa of cells from fused and patent cultured with OM. Donors N = 3; *p < 0.05; **p < 0.01; ***p < 0.001.

The results of the PCR array analysis regarding the effect of stiffness on the genetic expression of cells from patent and fused sutures is presented in the heatmaps (Fig. 5) for each individual donor, with red indicating stiffness-induced upregulation and green indicating stiffness-induced downregulation of the individual genes when cells were cultured in growth medium (Fig. 5A) or osteogenic medium (Fig. 5B). Despite the visible patient variability in the results, stiffness-dependent upregulation of ANGPT1 (p < 0.01) and BMP6 (p < 0.001) was observed in cells from fused sagittal sutures while AMOT, WIF1 and PRKCZ (p < 0.05), AMOTL2 (p < 0.01), AMOTL1, NOX1, JNK3, PRKCZ (p < 0.001) were upregulated in cells from patent coronal sutures when cultured with growth medium (Fig. 5C). Interestingly, when those cells were cultured with osteogenic medium the cells from fused sagittal sutures exhibited stiffness-dependent upregulation of IL1β, WIF1, BMP6 (p < 0.05), MMP9, NOX1, IGF1, and TSHZ2 (p < 0.001) in comparison to the expression in cells from patent coronal sutures (Fig. 5E). When comparing cells from patent sagittal sutures versus cells from fused sagittal sutures (Supplementary Figure S1), the stiffness-induced upregulation of genes was identical to the differences observed between coronal versus sagittal with the exception of the gene IL1β, which was not significantly different when comparing sagittal versus sagittal sutures. The PCR array results were further validated by real-time quantitative PCR for seven of the most highly expressed and relevant genes in craniosynostosis: when induced by stiffness and cultured with growth medium, expression of five genes increased in cells from fused sutures, BMP6, MSX2, TWIST and WNT2 (p < 0.05) and TGFβ, (p < 0.01) and no stiffness-mediated increases in gene expression were observed in cells from patent sutures (Fig. 5D). Finally, when induced by stiffness and cultured with osteogenic medium, expression of six genes increased in cells from fused sutures, BMP6, MSX2, TGFβ, TWIST (p < 0.05), JNK3 and WNT2 (p < 0.001) and no stiffness-mediated increases in gene expression were observed in cells from patent sutures (Fig. 5F). The stiffness-induced expression of FGFR3 was not statistically different between cells from patent and fused sutures but followed the same trend observed in the results of the PCR array. Our results indicate a stiffness-dependent increase in the expression of several genes associated with the osteoblastic bone matrix synthesis in cells from fused sutures.

## Discussion

Bone formation is a complex process that is guided by both biochemical and biophysical stimuli, including stimuli from the surrounding environment^26, 27, 30, 31^. During development, children have a remarkable capacity for bone healing compared to adults, due to their enhanced ability to repair and form new tissue. However, this process can sometimes be altered in developmental diseases, such as craniosynostosis, where an acceleration of ossification within patent sutures of the skull can prematurely form fused sutures. In assessing the causes for these alterations, previous studies have demonstrated an implication of abnormal mechanical cues in the pathogenesis of craniosynostosis, suggesting a role for altered force transmission in defining the timing and magnitude of premature suture fusion^21, 32^. The intrasutural mesenchyme is believed to contain undifferentiated and proliferative osteogenic stem cells that then differentiate into osteoprogenitor cells^14, 18, 33^. Moreover, a recent study identified Gli1^+^ MSC-like cells as the main stem cell population in the cranial sutures and being responsible for maintaining suture patency. Furthermore, this study suggested that a reduction in the number of stem cells may result in premature fusion of sutures^22^. For sutures to function as intramembranous bone growth sites, they need to remain in an un-ossified state, yet allow new bone to be formed at the edges of the overlapping bone fronts. This process relies on the production of sufficient new bone cells to be recruited into the bone fronts, while ensuring that the cells within the suture remain undifferentiated^20^. Therefore, the premature fusion of the sutures of children with non-syndromic craniosynostosis may be associated with changes in both the stimuli provided by the extracellular environment, as well as the functional capacity of cells within the sutures to sense and respond to the stimuli. We postulate that mechanical forces may activate the signalling cascades and alter osteogenic commitment and gene expression of the cells in the sutures.

Firstly, we characterised the cell populations obtained from patent and fused sutures in terms of their osteogenic potential by comparing them with fully differentiated osteoblasts. The ALP activity and mineralisation results showed similar calcium deposition by cells from patent and fused sutures when cultured in growth medium. Moreover, it is important to remark that the calcium deposition (Fig. 2B) of fully differentiated osteoblasts derived from calvarial bone cultured in growth medium was significantly higher than the levels observed in cells from patent and fused sutures. While further characterisation of the cell populations might be beneficial in the future in order to discern the specific composition of the cell population obtained from patent and fused sutures, results shown in Fig. 2 suggest that cells from fused sutures are not inherently different from cells from patent sutures in terms of their osteogenic commitment when cultured in standard *in vitro* tissue culture conditions. However, when cultured in osteogenic medium, cells from fused sutures expressed similar levels of calcium and ALP activity as fully differentiated osteoblasts and higher than that found in cells from patent sutures. This indicates that cells from fused sutures, when exposed to biochemical stimuli, are able to reach a more mature osteogenic phenotype faster than cells from patent sutures.

The cranial sutures, being less stiff than the surrounding calvarial bones, play pivotal mechanical roles resisting the tensile and compressive forces generated during skull development and these cells are subjected to different types of internal strains (for example, brain growth during development) and external strains (for example, intrauterine head constraint during childbirth)^34^. In order to understand the effect of biophysical cues directing suture patency, we cultured cells from patent and fused sutures on substrates of different stiffness. Cells from both patent and fused sutures increased their spreading area with increasing substrate stiffness as expected, which is consistent with results previously shown for osteoblasts as well as other cell types^26, 27, 35^. Stiffness affects the quality of cell attachment, which in turn affects the cell morphology initially, as well as their later proliferation and differentiation capability^36^. Here, we demonstrate that stiffness alone (i.e., without additional biochemical cues) induces changes in the morphology of cells from both patent and fused sutures. Interestingly, on substrates of identical stiffness, the differences in the morphology of cells from patent and fused sutures are visible after 48 hours in culture, in which cells from fused sutures (Fig. 3H) are more rounded and bigger than cells from patent sutures cultured on 100 and 300 kPa gels, resembling osteoblasts^35, 37^. In contrast, cells from patent sutures cultured on 300 kPa gels are elongated resembling the shape of mesenchymal stem cells (Fig. 3D). A previous study, quantifying the relationship between the different stages of osteoblast differentiation and cell morphology showed that size, shape, and traction all correlated with the differentiation stage of osteoblasts and, cell morphology evolved with differentiation^38^. Specifically, undifferentiated mesenchymal stem cell lines were small, spindly, and exerted low traction, while differentiated osteoblasts were large, had multiple processes, and exerted higher traction. Additionally, changes in the morphology of sutural cells due to the application of mechanical forces have also been demonstrated previously: application of cyclic, compressive loading to the cranial sutures causes changes in suture morphology, with an increase in suture width and sutural cell density of osteoblast-like and osteoclast-like cells^4^. Together, these facts corroborate our results showing that cells from fused sutures are reaching a more advanced stage of osteoblast differentiation than cells from patent sutures when subjected to physical cues.

As stiff substrates have also been shown to induce differentiation of mesenchymal stromal cells towards different lineages, including bone cartilage, muscle and fat (Engler *et al*.^26^), we further investigated whether stiffness is a biophysical cue capable of triggering mineralisation in cells from the sutures of the calvarial of children with non-syndromic craniosynostosis and if this stimulus (i.e., changes in the stiffness of the microcellular environment) could have potential implications in premature suture fusion. We assessed mineralisation and observed no statistical differences in calcium deposition between cells from patent and fused sutures on soft substrates (10 kPa). However, there was an increase in mineralisation by cells from fused sutures with increasing stiffness but not by cells from patent sutures (Fig. 4A), indicating that stiffness induces mineralisation by cells from fused sutures. Interestingly, with the addition of osteogenic medium, we observed an enhanced sensitivity for both cells from patent and fused sutures to stiffer substrates (Fig. 4B). This suggests a stiffness-induced craniosynostosis, in which stiff substrates may trigger premature fusion of the sutures by promoting an accelerated osteogenic commitment of the cells from patent sutures. Additionally, the increase in mineralisation was greater in cells from fused sutures than in cells from patent sutures, which might be due to their higher sensitivity and quicker commitment to a more advanced stage of osteogenic differentiation. In line with our experiments, early ALP activity was detected at the sagittal suture line, indicating a front of developing bone in preparation for fusion after 14 days of *in vitro* cyclical, compressive load^20^. Moreover, another study indicated that after 14 days of applied mechanical loading, type I collagen and calcified bone matrix appeared at the edge of the craniofacial sutures^39^. These findings support our findings regarding the induction of new bone formation in the sutures in response to mechanical force.

To date, most research studies on suture fusion have focused on genetic mutations^17, 33, 40, 41^. Contrastingly, in this study we analysed changes in the gene expression of several genes associated with patency and premature fusion of the sutures when subjected to different substrate stiffness. Using a PCR array, we identified stiffness-induced upregulation of ANGPT1 and BMP6 in cells from fused sutures when cultured with growth medium and WIF1, NOX1, BMP6, TSHZ2, IGF1, MMP9 and IL1β in cells from fused sutures when cultured with osteogenic medium. Using qPCR analysis; we further showed the stiffness-induced upregulation of BMP6, JNK3, MSX2, TGFβ, TWIST and WNT2 in cells from fused sutures. In particular, IL1β expression, which is one of the most relevant pro-inflammatory cytokines, has been identified in several bone degenerative diseases, such as osteoarthritis^42^. Moreover, IL1β has been also associated with the promotion of the cartilage breakdown, the downregulation of the genetic expression of the extracellular matrix components as well as the increased synthesis of proteolytic enzymes including several metalloproteinase (MMP)-1, -3 and -9, which are enzymes responsible for breaking down the extracellular matrix, processes that were followed by endochondral ossification^42^. Our PCR array results revealed an overexpression of BMP6 (also confirmed by qPCR) in cells from fused sutures when cultured in growth and osteogenic medium, while the expression of osteogenic markers such as RUNX2, BMP2, SMADS, among others members of the RUNX2/BMP2 signalling pathway were downregulated by stiffness regardless the culture medium (Fig. 5A,B). Previous publications have pointed out that IL1β alone or in combination with TNFα is able to block the BMP2-dependent osteogenic differentiation by inhibiting the RUNX2 activation^43^. It has also been shown a BMP6-dependent increase of osteoblastic differentiation in RUNX2^−/−^ calvarial-derived mesenchymal cells, suggesting that BMP6 pathway might be an alternative pathway for bone formation, independent on the activation of the RUNX2/BMP2 pathway^44^. Moreover, the expression of NOX1 and the subsequent increase of reactive oxygen have been associated with the activation of RANKL and osteoclast differentiation^45, 46^. While further studies would be necessary in order to clarify the signalling pathways that are controlling the premature ossification of the fused sutures, our results together with previous findings suggest that the stiffness-induced increase in the osteogenic response of cells from fused sutures might happen through the activation of the BMP6 signalling pathway.

We also identified the stiffness-dependent activation of insulin growth factor that plays an essential role in skeletal development in cells from fused sutures. IGF1 is an essential growth factor for bone formation and it has been demonstrated that an increase in the production of IGF1 and the activation of IGF1R at the ossification sites are also key modulators of the extracellular cartilage calcification^47^. TSHZ2 is another gene involved in the craniofacial skeletal development and alteration of its activity has been linked to craniofacial deformities, including the collapse of the craniofacial structures due to insufficient bone and cartilage formation^48^. Additionally, silencing TSHZ2 in mice resulted in loss of neural crest–derived cells, which are present in the cranial sutures, revealing the key role of TSHZ2 in the craniofacial bone development^49^. In our work we observed an augmented expression of IGF1 and TSHZ2 due to stiffness in cells from fused sutures and given their role in bone formation, this suggests that the over expression of these genes in cells from fused sutures may have an important role in the accelerated bone formation.

Moreover, Behr *et al*. showed that canonical WNT signalling also plays a key role in suture fate^16^, in which closure of the suture was found to be accompanied by a downregulation of canonical WNT signalling, whereas suture patency was associated with constitutively activated canonical WNT signalling. In our results we see stiffness-dependent upregulation of WIF1 in cells from fused sutures, an inhibitor of the canonical WNT pathway therefore, which leads to suture fusion. WNT2 expression may happen through activation of either canonical WNT pathway (β-catenin-dependent) or non-canonical WNT pathways, including the MAPK non-canonical pathway, which involves the activation of Rho, ROCK and JNK3^50, 51^. The activation of this pathway may be dependent on the mechanical stimuli and modulates cell shape and fate^52^. In our study, we observe stiffness-induced expression of JNK3 and WNT2 as well as activation of the WIF1, the inhibitor of the canonical pathway in cells from fused sutures. This suggests not only that WNT2 may be act in these cells through the non-canonical WNT pathway but also that accelerated osteogenesis is dependent on the activation of a mechano-regulated pathway through the activation of JNK3, which has been previously associated to stiffness activated osteoinduction in children-derived MSCs^53^. Consequently, we demonstrated that the accelerated bone formation in cells from fused sutures associated with craniosynostosis is linked with the stiffness-dependent activation of the MAPK-associated non-canonical WNT pathway through activation of JNK3 and WNT2.

In this study cells from patent sutures were obtained from patients with non-syndromic craniosynostosis. A previous study has shown that gene expression observed for the patent sutures of patients with craniosynostosis was not significantly different from the expression presented by patent sutures of patients without the disease^54^. This suggests that the cell behaviour of patent sutures from patients with craniosynostosis, may provide valuable information that helps to understand better what happens in patent sutures from healthy children.

In conclusion, we have identified that cells from fused sutures have greater expression of osteogenic markers when stimulated with biochemical and/or biophysical cues, confirming the accelerated bone formation in fused versus patent sutures. Additionally, this study highlights the differences in mechanoresponsiveness between cells from fused and patent sutures, showing a stiffness-dependent increase in osteogenesis and in mineralisation by cells from fused sutures, earlier than in cells from patent sutures. Moreover, we identified the mechano-pathways involved in suture fate thus, demonstrating that a connection between stiffness-induced gene expression and craniosynostosis may exist. Stiff substrates induce activation of genes involved in the breakdown of the extracellular matrix, activation of inflammation and bone formation in cells from fused sutures but not in cells from patent sutures. Together, these results suggest that stiffening of the extracellular environment may trigger the premature ossification of the sutures. Our results further suggest that craniosynostosis may be linked to an abnormal mechanical environment, suggesting a role for altered force transmission in defining the timing and magnitude of premature suture fusion. Understanding the changes in regulation of the genes associated with suture patency may open up avenues to further identify the potential mechanotransductive mechanisms associated with craniosynostosis and for the development of therapeutic strategies to rescue prematurely fusing sutures.

## Methods

### Cell isolation

Tissue samples from patent sutures, fused sutures and normal calvarial bone of children with craniosynostosis were collected during cranial vault remodelling procedures at the National Paediatric Craniofacial Centre, Temple Street Children’s University Hospital, Dublin in 50 mL falcon tubes containing PBS at room temperature. Informed written parental consent was obtained prior to our investigations and all methods were performed in accordance with the relevant guidelines and regulations (ethical approval n°. 13022 from the Temple Street Children’s University Hospital). All calvarial samples were obtained from discarded tissues during the surgical reconstructive procedure from patients 5 to 28 months old and non-syndromic (Table 1). In sterile conditions, the three different sample groups were washed thoroughly with PBS (Sigma-Aldrich, Ireland) using a cell 70 µm strainer attached to a 50 mL falcon tube to collect the transporting liquid and the washed cells were then transferred into new 15 mL falcon tubes. Samples were centrifuge at 400 * g for 5 minutes and the supernatant was removed. Cells were extracted from the bone and suture tissue samples using a freshly prepared digestion buffer consisting of 300 Units/mL of collagenase Type F (Sigma-Aldrich, Ireland) and 0.25% trypsin (Sigma-Aldrich, Ireland)^55, 56^. Five sequential digestions (I–V) were performed at 37 °C for 10, 20, 30, 50 and 70 minutes^57^, respectively using 1 mL of the digestion buffer into each sample tube. After each digestion, bone samples and cells were centrifuge at 400*g for 5 minutes. During digestions I and II all the cells were discarded in order to avoid a cell population containing blood cells. The bone samples were again washed with PBS in a 40 µm cell strainer and transferred into the same 15 mL falcon tube with 1 mL of the digestion buffer ready for the next digestion. From digestion III-V cells were pooled together into a 50 mL tube^58^, washed with PBS and centrifuged twice at 400*g for 5 minutes before being re-suspend into 5 mL of growth medium, composed of low glucose Dulbecco’s Modified Eagles medium (DMEM) supplemented with 10% foetal bovine serum (FBS) and 1% penicillin/streptomycin (P/S) (Sigma-Aldrich, Ireland), and seeded into T25 flasks, while the digested bone samples were discarded. A complete media change was done on the fifth day and after that, every three days. Upon confluence, cells were passaged and used in the following experimental setups. This procedure allowed the isolation of osteoprogenitor cells contained in the patent and fused sutures as well as the fully differentiated osteoblast cells contained in the calvarial bone.

**Table 1:** Location and state of sutures and normal calvarial bone obtained from children with non-syndromic craniosynostosis used in the different experimental setups.

Analysis	Patient #	Sex	Patent Suture	Fused Suture	Normal Calvarial
*Characterisation of osteogenic population*	2	M	Coronal	Sagittal	Parietal
	4	M	Coronal	Sagittal	Parietal
	6	F	Coronal	Sagittal	Parietal
*Effect of stiffness in cell morphology*	2	M	Coronal	Sagittal	N.A.
	4	M	Coronal	Sagittal	N.A.
	5	M	Coronal	Sagittal	N.A.
*Effect of stiffness in osteogenesis*	1	F	Sagittal	Coronal	N.A.
	2	M	Coronal	Sagittal	N.A.
	3	M	Coronal	Sagittal	N.A.
*PCR array*	1	F	Sagittal	Coronal	N.A.
	2	M	Coronal	Sagittal	N.A.
	4	M	Coronal	Sagittal	N.A.
	5	M	Coronal	Sagittal	N.A.
*qPCR*	1	F	Sagittal	Coronal	N.A.
	2	M	Coronal	Sagittal	N.A.
	4	M	Coronal	Sagittal	N.A.
	5	M	Coronal	Sagittal	N.A.

### Assessment of osteogenic potential of cells from patent and fused sutures

Cells isolated from patent and fused sutures patients with non-syndromic craniosynostosis were characterised in terms of their phenotype and potential for osteogenic differentiation (passages 3–4). Cells from patent and fused sutures were cultured at a density of 3125 cells/cm^2^ in 6-well plates in both growth medium (GM) and osteogenic medium (OM) to analyse their osteogenic potential and phenotype by comparing them with fully differentiated osteoblasts from normal calvarial bone cultured at the same density and used as a control for cell differentiation. These cells were obtained from the calvarial bone in locations remote from the sutures of children with non-syndromic craniosynostosis (disease that affect the physiology of the cells within the sutures) and therefore, these cells are considered as physiologically normal osteoblasts, not affected by the disease state of the patients. The osteogenic media consisted of growth media supplemented with osteogenic factors, including 100 nM dexamethasone, 50 µg/mL ascorbic acid and 10 mM β-glycerophosphate (Sigma Aldrich, Ireland). The osteogenic potential of the different cell samples was analysed in terms of alkaline phosphatase (ALP) activity (ANASPEC) after 7 days in culture and calcium deposition in the extracellular matrix (StanBio) after 14 days in culture, according to the manufacturer’s protocol and used as the key differentiation markers in assessing expression of the osteoblast phenotype. ALP activity and mineralisation results were normalised by measuring the dsDNA content per sample using the Quant-iT PicoGreen dsDNA kit (BioSciences, Ireland).

### Collagen-coated polyacrylamide substrates fabrication and cell seeding

To assess the changes in the mechanosensitivity of cells from CS patients, polyacrylamide (PAA) gels were produced by mixing different ratios of 40% acrylamide and 2% bis-acrylamide monomer concentrations in _dd_H_2_O, and inducing free radical polymerisation using ammonium persulfate (APS) and tetramethylethylenediamine, following a methodology described in literature^59^. Briefly, polymerisation was performed between glass slides in an oxygen-free environment, using 32 mm glass coverslips that was subjected to a plasma treatment for 3 min and to a methacrylate solution (Sigma-Aldrich, Ireland) to assure a clean surface for gel adhesion. The other glass surface was treated with chlorotrimethylsilane (Sigma-Aldrich, Ireland), which makes the glass surface hydrophobic to allow gel detachment without breaking the gel. PAA gels of 1, 10, 100 and 300 kPa were produced and collagen type I solution from rat tail (50 µg/mL) was covalently bound to the surface of the gels at 37 °C overnight after coating the gel with 0.2 mg/mL of sulfo-SANPAH (Proteochem, USA) diluted in PBS under 365 nm UV lamp for 30 minutes, which was also used as a sterilisation step. Stiffness of the substrates was characterised by mechanical testing with a rheometer (TA Instruments, USA) and gel thickness of 1 mm was measured using a Dektak profilometer. The collagen-coated PAA substrates were washed twice with PBS before seeding with cells from patent and fused sutures at different densities depending on the assay. All the cells were seeded using GM for 24 hours to allow adhesion before replacing with fresh GM or OM.

### Mineralisation

Functional expression of osteogenic markers was measured in cells from patent and fused sutures at a density of 3125 cells/cm^2^ on 10 (soft) and 300 (stiff) kPa collagen-coated PAA substrates in control medium (GM) or OM. The effect of stiffness of the osteogenic potential of these cells was assessed by measurement of calcium deposition in the extracellular matrix (StanBio) after 14 days in culture, according to the manufacturer’s protocol. Mineralisation results were normalised by measuring the dsDNA content per sample using the Quant-iT PicoGreen dsDNA kit (BioSciences, Ireland).

### Morphology study

The effect of stiffness on cell morphology was evaluated by measuring cell-spread area of cells from patent sutures and cells from fused sutures cultured at 1000 cells/cm^2^ on 1, 10, 100 and 300 kPa PAA substrates after 48 hours in culture. Measurements of the area of cells were performed on images taken using a 10X objective in a Leica microscope, using analyse particles menu in ImageJ to measure cell area of twenty-one cells, seven per donor.

### PCR array

Cells from patent and fused sutures were seeded at a density of 7000 cells/cm^2^ onto the 10 (soft) and 300 (stiff) kPa substrates in both GM and OM for 7 days after which the RNA was isolated using an RNeasy Minikit (Qiagen) following the manufacturer instructions. Briefly, reverse transcription of the samples was performed with RT2 First Strand Kit (Qiagen) followed by plating 0.5 μg cDNA and RT2 SYBR Green Master Mix (Qiagen) in the array plates. The list of primers used for the PCR array study was custom designed (Qiagen) as previously described^53^, containing osteogenesis-, angiogenesis-, mechanotransduction- and craniosynostosis-related genes in addition to five housekeeping control genes, one human genomic DNA contamination control, two RT-controls and two positive PCR control. For the PCR array cycle, amplification was performed with a 10 minutes 95 °C activation step, followed by 40 cycles at 95 °C for 15 seconds (denaturation) and 1 minute at 60 °C (extension). The fold induction for each gene was normalised by the expression of the RPL0 –housekeeping- of each sample and analysed by the ΔΔCt method. Results are presented as the fold induction between the genetic expressions of cells cultured on stiff vs soft substrates. The heatmaps were obtained from the online version of the Matrix2png software.

### Quantitative real-time polymerase chain reaction

mRNA was isolated at day 7 from cells from patent and fused sutures cultured on 10 and 300 kPa collagen-coated substrates in OM, using the RNeasy Minikit (Qiagen) according to the manufacturer’s instructions. Briefly, two-step reverse transcription and real-time PCR were performed using Quantitect Reverse Transcription Kits and Quantitect SYBR Green PCR Kits (Qiagen), respectively, loading 2.5 ng of cDNA per reaction. Gene expression was analysed using the same four RNA samples which underwent PCR array analysis. Primer amplification efficiency was compatible with the comparative Ct method used for expression of the results. The fold induction index was normalised by the housekeeping expression of each sample to compare the genetic expression of cells cultured in stiff substrates against the expression in soft substrates. The primers used for the real time PCR were: JNK3 (Qiagen, QT00006923); MSX2 (Qiagen, QT00015295); TGFβ (Qiagen, QT00000728); BMP6 (Qiagen, QT00034846); WNT2 (Qiagen, QT00022071); TWIST (Qiagen, QT00011956); FGFR3 (Qiagen, QT01000685) and; housekeeping 18S (Qiagen, QT00199367). The PCR was initiated with an activation step of 15 minutes at 95 °C, followed by 40 cycles of denaturation (15 seconds, 94 °C), annealing (30 seconds, 55 °C) and extension (30 seconds, 72 °C), followed by the melting curve as recommended by the manufacturer, in an Eppendorf® Mastercycler® ep realplex 4.

### Statistical analysis

All experiments were performed for 3 donors, each containing 3 replicates per stiffness conditions and 3 replicates per medium conditions. Data analysis was performed using the GraphPad Prism software package. Results were expressed as the mean ± SEM. Statistical significance was determined using a two-way analysis of variance (ANOVA) followed by a post hoc Bonferroni’s correction (p < 0.05).

## Electronic supplementary material

Supplementary Figure S1
