# Supplementary material for: Identification of stiffness-induced signalling mechanisms in cells from patent and fused sutures associated with craniosynostosis
Source: Sci Rep. 2017 Sep 13;7:11494. doi: 10.1038/s41598-017-11801-0 (PMC5597583; doi:10.1038/s41598-017-11801-0)
Supplement: Supplementary file 1 — Supplementary Figure S1 [file 41598_2017_11801_MOESM1_ESM.pdf]

Identification of stiffness-induced signalling mechanisms in cells from patent and fused sutures associated with craniosynostosis

Sara Barreto[1,2,3*]; Arlyng Gonzalez-Vazquez[,1,2,3*]; Andrew Cameron[1,2,3]; Fergal J. O'Brien[1,2,3,#] and Dylan J. Murray[4,#]

[1]Tissue Engineering Research Group (TERG), Department of Anatomy, Royal College of Surgeons in Ireland (RCSI), Dublin2, Ireland; [2]Trinity Centre for Bioengineering, Trinity College Dublin (TCD), Dublin 2, Ireland; [3]Advanced Materials and Bioengineering Research (AMBER) Centre, CRANN Institute, Trinity College Dublin, Dublin 2, Ireland; [4]National Paediatric Craniofacial Centre, Temple Street Children's University Hospital, Dublin 1, Ireland.

*Joint-first authorship; # Joint-last authorship

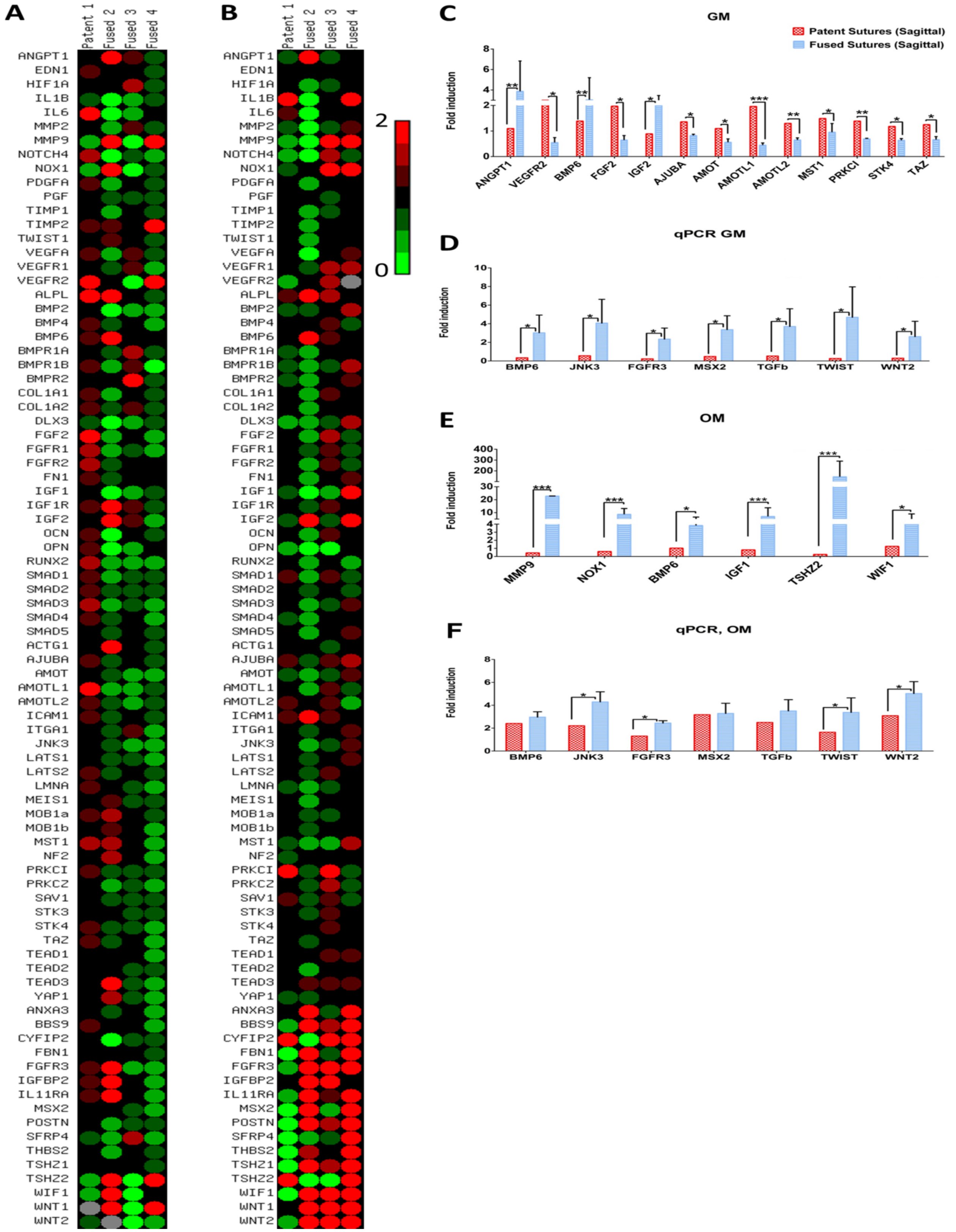

Supplementary Figure S1: Stiffness-induced gene upregulation of cells from fused and patent sagittal sutures cultured for 7 days on soft (10 kPa) and stiff (300 kPa) substrates. (A) Heatmaps of the genetic expression of cells from patent and fused sutures representing the fold induction from cells cultured with growth medium (GM) on stiff to soft substrates, per donor. (B) Heatmaps of the genetic expression of cells from patent and fused sutures representing the fold induction from cells cultured with osteogenic medium (OM) on stiff to soft substrates, per donor. (C) Statistically significant stiffness-dependent gene upregulation of cells from fused and patent sutures cultured in GM and presented as the average fold induction of cells cultured on stiff and soft substrates. (D) Selection of genes validated by qPCR comparing fold induction from stiff to soft of cells from fused and patent sutures cultured in GM. Donors N = 3; *p<0.05; **p<0.01; ***p<0.001.
